# The New Era of Biologics in Atopic Dermatitis: A Review

**DOI:** 10.5826/dpc.1104a144

**Published:** 2021-10-01

**Authors:** Simon Schneider, Linda Li, Alexander Zink

**Affiliations:** 1 Technical University of Munich, School of Medicine, Department of Dermatology and Allergy, Munich, Germany; 2 Division of Dermatology and Venereology, Department of Medicine Solna, Karolinska Institute, Stockholm, Sweden

**Keywords:** atopic dermatitis, therapy, biological, dupilumab

## Abstract

Atopic dermatitis (AD) is a prevalent inflammatory skin disorder affecting all age and ethnic groups. The age-dependent varying appearance and extent of pruritic lesions are accompanied by distinct individual suffering, highlighting the importance of effective treatment options. Over the past years systemic drugs have considerably extended therapeutic approaches of patients with moderate to severe AD, in particular new biologics, most notably dupilumab has appeared as major breakthrough. In addition to monoclonal blockade of IL-4 and IL-13 pathway, more cytokines have been found to play a substantial role in AD pathogenesis, presenting potential targets for new therapy options.

## Introduction

Atopic dermatitis (AD), one of the most common inflammatory skin disorders affecting people of all ages and ethnicities, is characterized by typical age-dependent clinical features and substantial individual suffering as well as extensive economic impact [1, 2]. Whereas infants often present acute eczematous pruritic lesions involving the face, cheeks, and trunk, children, adolescents, and adults develop rather diffuse lesions affecting the flexures, alternating between acute and chronic areas [3].

With numerous new emerging drugs expanding the clinical practice of AD healthcare in recent years, the approval of dupilumab, represented a major breakthrough in the therapy of patients with moderate to severe AD. This review aims to describe currently available biological treatment options and potential future developments.

### Dupilumab

The pathophysiology of AD is multifactorial, involving a genetic predisposition, epidermal barrier dysfunction, the skin microbiome, and type-2-T-helper-cells (Th2)-predominant inflammation [4–6]. The latter is mediated by various type-2 cytokines, among others interleukin-4 (IL-4), and IL-13. Moreover, IL-4 induces differentiation of Th-cells into Th2-cells, thus promoting further production of IL-4 and IL-13 [7]. Both using the same IL-4 receptor subunit alpha (IL-4Rα), these cytokines are responsible for IgE class switching in B-cells [8]. Given its key role in the pathway of type-2 mediated immune response, IL-4Rα blockade was anticipated to represent a therapeutic approach to treating allergic diseases. Indeed, dupilumab, a human monoclonal antibody blocking IL-4Rα, was first shown to be effective in the treatment of AD in an early-phase randomized, double-blind, placebo-controlled trial in adults in 2014 [9]. Monotherapy of dupilumab at 4 weeks compared to placebo showed a rapid and dose-dependent 50% improvement in the Eczema Area and Severity Index score (EASI-50) and decline in the pruritus numerical-rating scale. This effect was further intensified at 12 weeks [9]. Dupilumab was previously shown to be efficient in treating persistent, moderate-to-severe asthma with elevated eosinophil levels [10]. Simpson et al reported in 2016 an improvement of 75% in the EASI, a reduction of pruritus and improvement in quality of life in two phase-3 trials of dupilumab versus placebo in AD, comprising almost 1,400 patients aged 18 years and older in North America, Europe, and Asia.

Adverse effects were mild to moderate and comprised nasopharyngitis, upper respiratory tract infections, conjunctivitis, injection-site reactions, and exacerbation of AD [11]. In 2017, dupilumab was authorized for the treatment of AD in the European Union by the European Medicines Agency (EMA). Shortly after, phase-3 trials for adolescents [12] and children aged 6 years and older [13] were conducted, which showed an improvement in EASI, Investigator’s Global Assessment (IGA) score, and quality of life compared to placebo with similar adverse effects in these age groups as well. Currently, the treatment of AD with dupilumab is authorized by EMA for patients aged 6 years and older. Recently, a promising phase-2, two-age cohort, two-dose level, multicenter study of dupilumab in the treatment of severe uncontrolled AD in children aged 6 months to < 6 years has showed efficacy and a similar safety-profile as seen in older age groups [14]. These data could support a phase-3 trial of dupilumab in this patient population to further offer therapeutic approaches for topical corticosteroid-refractory AD in this age group.

### Further Biologics in Atopic Dermatitis’ Treatment

In addition to IL-4 and IL-13, other cytokines have been identified to play an important role in the pathophysiology of chronic inflammatory skin disease. These are IL-31, thymic stromal lymphopoietin (TSLP), IL-17, and IL-22 cytokines, which can also negatively affect the expression of barrier proteins.

#### IL-13

A Th-2 mediated exuberant immune response is stated to be the key mechanism in the pathogenesis of AD. Zheng et al induced pruritic dermatitis and skin remodeling in an IL-13 overexpressing mouse model. They showed that IL-13 was mainly produced in the skin and caused xerosis, itching lesions, chronic inflammation of the skin, and dermal infiltration of CD4^+^-cells, mast cells, and eosinophils. Mice models’ skin also showed increased fibrosis and vascularization [15]. Another study was able to show a relative upregulation of IL-13 mRNA compared to IL-4 mRNA in lesional skin of AD patients, thus providing first evidence that IL-13 may be the crucial cytokine in the IL-4/IL-13 axis [16]. IL-13 as a potent stimulator of dermal inflammation and remodeling is another hypothesis for the pathogenesis of AD [17, 18].

Both lebrikizumab and tralokinumab, monoclonal antibodies that target IL-13, have been recently investigated for AD treatment. The effectiveness of a therapy with Tralokinumab was analyzed in two 52-week, randomized, double-blind, multicenter, placebo-controlled phase-3-trials among more than 1,500 participants. Tralokinumab showed significant reduction in EASI and IGA scores in a dose-dependent manner compared to placebo. Participants receiving tralokinumab had a response rate of up to 70% for EASI-50 and up to 40% for EASI-75. In addition, early improvements were observed in pruritus, sleep disturbances, dermatology life quality index (DLQI), and the severity scoring of atopic dermatitis (SCORAD). Those effects were demonstrated in most participants at week 16, and after extended use, at week 52. The most common adverse reactions due to tralokinumab use were upper respiratory tract infections, conjunctivitis, and injection site reactions [19]. Based on these study results, the EMA recommended tralokinumab approval for the treatment of moderate to severe AD, in June 2021.

Like tralokinumab, lebrikizumab is an IL-13 antibody. Guttman-Yasky et al examined the effects of the biological lebrikizumab in a double-blind, placebo-controlled, 16-week, phase-2b study among 280 individuals who were randomly assigned to a placebo group or to groups receiving lebrikizumab in different doses. A rapid dose-dependent effect of lebrikizumab was described in clinical scores and symptoms when compared to placebo. For instance, after 16 weeks, an EASI improvement of up to 72.1% was shown in the group receiving 250 mg lebrikizumab every 2 weeks compared to placebo (P < 0.001). Adverse events were mainly injection site reactions, herpes virus infections, and conjunctivitis. No adverse events led to premature patient discontinuation. If the positive effect of this biological can be reproduced in currently underway phase-3 studies, lebrikizumab can be considered a highly effective therapy modality in the treatment of moderate to severe AD [20].

#### IL-31

Intense itching is one of the greatest burdens of AD patients. IL-31, a cytokine primarily produced by CD4+ T-cells, has been found to be increased in skin samples of AD patients compared to healthy subjects [21]. Takamori et al studied IL-31-deficient mice models. Interestingly these showed decreased scratch frequency and duration, during induced contact dermatitis [22]. Treatment with IL-31-antibodies also showed reduced scratching behavior in AD-induced NC/nga mice [17, 18, 23, 24].

In a randomized, double-blind, placebo-controlled study, Ruzicka et al investigated the safety and efficacy of nemolizumab, a humanized antihuman IL-31a receptor antibody, in combination with topical steroids in patients with AD. The results showed that subcutaneous administration of Nemolizumab was well tolerated by all subjects. Nemolizumab significantly decreased pruritus compared to placebo (P < 0.01), but no significant reduction in EASI was observed. Accordingly, nemolizumab appears to be another therapeutic option, especially for AD patients suffering from pruritus [24, 25]. Currently, there are 3 ongoing clinical phase-3 studies to prove nemolizumab safety and efficacy among a larger number of patients suffering from AD (NCT03989349, NCT03989206, NCT03985943, clinicaltrials.gov).

#### Thymic Stromal Lymphopoietin and OX40

The TSLP is a cytokine that is upregulated by IL-13 and directly leads epidermic dendritic cells (DC) to a Th-2-response. TSLP is mainly produced in keratinocytes and appears to play a key role in the activation of DC. Due to allergen damage, keratinocytes express TSLP, which in turn activates DC, thus increasing their expression of OX40L. OX40L leads to the differentiation of naive CD4^+^-cells into inflammatory Th-2-memory cells. OX40L is expressed by DC and activates T-cells and memory cells. The interaction between OX40 and OX40L appears to be critical for the long-term survival of CD4^+^ T-cells, which are responsible for inflammation in AD [17, 18].

There is evidence that mice overexpressing TSLP develop AD and that their lesional skin contains increased numbers of Th-2 cells. Current studies are investigating whether this pathway can be used for new therapeutic options for the treatment of AD. Guttman-Yassky et al investigated the efficacy and safety of GBR830, a humanized antibody against OX40, in an explorative phase-2a, placebo-controlled study among patients with moderate to severe AD. First results showed that GBR380 was well tolerated and showed a significant reduction in Th1-, Th2, and Th17/22 expression in lesional skin compared to placebo (P < 0.01). Furthermore, a significant reduction in the epidermal thickness could be observed (P < 0.001)[26]. The phase-2b trial is completed, but results have not yet been published (NCT03568162).

A phase-2a study from Japan, which also showed promising results, investigated KHK4083, a monoclonal antibody against OX40. A continuous reduction of EASI and IGA scores was achieved in this study. Furthermore, KHK4083 showed an acceptable safety profile [27].

Tepezelumab, a monoclonal antibody, is another medication targeting TSLP. In a phase-2 study, 113 patients were 1:1 randomized and treated either with placebo or subcutaneous Tepezelumab every 2 weeks. Results of this trial showed that a higher percentage of patients treated with Tepezelumab reached an EASI50 after 12 weeks compared to the placebo group; however, the effect was not significant (P = 0.91) [28]. To show possible beneficial effects of this treatment option, larger studies need to be conducted.

#### IL-33

IL-33 is another key cytokine involved in AD pathogenesis. Increased amounts of IL-33 have been detected in AD patients’ lesional skin as well as in mice models. Furthermore, IL-33 has been shown to induce Th-2 immune response and significantly promote the release of IL-4, IL-5, and IL-13 as well as increase the activity of OX40L. Blocking IL-33 and subsequently suppressing the mentioned cytokines could also be leveraged in the therapy of AD [29–31]. The first effects of an IL-33 inhibition were showed in a mouse model [32].

In a phase-2a study, Chen et al examined the in-vivo effect of etokimab, (ANB020), a monoclonal IgG-antibody, in 12 patients with moderate to severe AD. After a single systemic administration of etokimab, 83% of patients achieved EASI-50 and 33% EASI-75 at day 29. A significant reduction in neutrophile infiltration of the skin was also observed compared to placebo. These results suggested that IL-31 suppression can positively affect inflammatory responses and may represent another component of AD therapy [33]. A phase-2 trial has been initiated and is currently in the recruiting phase (NCT03533751).

#### TH-22/IL-22

AD is traditionally considered a Th-2-mediated disease. However, it has previously been shown that Th-22 cells also play an important role in pathogenesis. Th-22 cells express IL-22, which in turn activates a receptor responsible for epidermal hyperplasia, migration of keratinocytes, downregulation of keratinocytic differentiation, and elevation of proinflammatory cytokines. In vitro analyses of skin biopsies of patients showed a correlation between the severity of AD with the presence of CD8^+^-IL-22-cells [17, 34, 35].

Guttmann-Yasky et al performed a randomized, double-blind, placebo-controlled trial testing the efficacy and safety of fezakinumab, a monoclonal IgG-antibody against IL-22. A significant reduction in the SCORAD (≥ 50) was found in the subgroup of patients with severe AD compared to placebo (P < 0.029), but this effect was not found for the entire study population. At week 12, a significant reduction of the body surface area involvement was observed in all patients receiving fezakinumab compared to placebo. In addition, fezakinumab demonstrated a beneficial safety profile, with upper respiratory tract infections being the most reported adverse event, which will require further investigation in a larger study population [36].

## Conclusion

**Figure 1:**
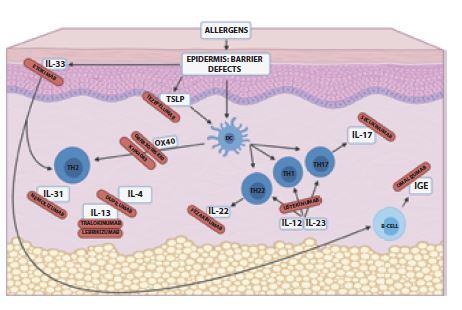
Pathogenesis and therapeutic targets in atopic dermatitis (AD). Modified according to Worm et al [35] and Li et al [14]. Increased skin penetration of allergens due to epidermal barrier defects results in type-2-T-helper-cells (Th2)-predominant inflammation with and without activation of dendritic cells (DC). Various cytokines contribute in the pathogenesis of AD, representing effective and future possible therapeutic targets for biologics in combating AD. Cells that activate DC or those that are activated by them, such as TSLP or OX40, represent potential targets.

New findings on the pathogenesis of AD introduce new therapeutic approaches almost daily as summarized in Table 1 and Figure 1. For example, the Th-17/IL-23 axis, which was long considered pathognomonic for psoriasis, could now also be proven to play a role in the pathogenesis of AD [37]. Already proven agents for the therapy of psoriasis are available and may be used for therapeutic approaches for patients suffering from AD. Described findings and clinical results illustrate the viability of biologicals and support the endeavor of targeted, patient-specific medication for the treatment of AD.

## Figures and Tables

**Table 1:** Overview of Biologicals Approved or Tested for Atopic Dermatitis Treatment.

Biological	Target	Current phase of clinical trials	Main findings in clinical trials
Dupilumab	IL-4/IL-13	Approved for clinical use by EMA	EASI-75 improvement after 16-weeks of trial (Phase III) [11]
Tralokinumab	IL-13	Approved for clinical use by EMA	EASI-75 improvement & IGA 0 or 1 after 16-weeks of trial compared to placebo (Phase III) [19]
Lebrikizumab	IL-13	3, still recruiting	Up to 72.1% improvement EASI (250mg dose) after 16-weeks of trial compared to placebo (Phase IIb) [20]
Nemolizumab	IL-31	3, still recruiting	Up to 63.1% change in the pruritus VAS score after 12-weeks compared to placebo (Phase IIb) [24]
GBR830/IBS830	OX40	2b, finished, results not published	EASI-50 improvement after 71 days compared to placebo (Phase IIa) [26]
KHK4083	OX40	2a, completed, results not published	Continued improvement in EASI and IGA (Phase I) [27]
Tepezelumab	TSLP	2b, still recruiting	Numerical EASI50 improvement after 12-weeks of trial compared to placebo (Phase IIa) [28]
Etokimab	IL-31	2b, still recruiting	83% improvement of EASI50 and 33% EASI75 after 29-days of a single dose (Phase IIa) [33]
Fezakinumab	IL-22	2a, completed	SCORAD improvement greater compared to placebo at 12-weeks of trial (Phase IIa) [36]

* Data from clinicaltrials.gov, Worm et al [18], & Li et al [17].
